# Facile Synthesis of SiO_2_@C Nanoparticles Anchored on MWNT as High-Performance Anode Materials for Li-ion Batteries

**DOI:** 10.1186/s11671-017-2226-2

**Published:** 2017-07-18

**Authors:** Yan Zhao, Zhengjun Liu, Yongguang Zhang, Almagul Mentbayeva, Xin Wang, M. Yu. Maximov, Baoxi Liu, Zhumabay Bakenov, Fuxing Yin

**Affiliations:** 1 School of Materials Science and Engineering, Research Institute for Energy Equipment Materials, Hebei University of Technology, Tianjin, 300130 China; 2 Synergy Innovation Institute of GDUT, Heyuan, Guangdong Province China; 3 Institute of Batteries LLC, National Laboratory Astana, School of Engineering, Nazarbayev University, 53 Kabanbay Batyr Avenue, Astana, 010000 Kazakhstan; 4 Peter the Great Saint-Petersburg Polytechnic University, Saint Petersburg, 195221 Russia

**Keywords:** Lithium-ion battery, Anode, SiO_2_@C/MWNT composite, Sol-gel synthesis

## Abstract

Carbon-coated silica nanoparticles anchored on multi-walled carbon nanotubes (SiO_2_@C/MWNT composite) were synthesized via a simple and facile sol-gel method followed by heat treatment. Scanning and transmission electron microscopy (SEM and TEM) studies confirmed densely anchoring the carbon-coated SiO_2_ nanoparticles onto a flexible MWNT conductive network, which facilitated fast electron and lithium-ion transport and improved structural stability of the composite. As prepared, ternary composite anode showed superior cyclability and rate capability compared to a carbon-coated silica counterpart without MWNT (SiO_2_@C). The SiO_2_@C/MWNT composite exhibited a high reversible discharge capacity of 744 mAh g^−1^ at the second discharge cycle conducted at a current density of 100 mA g^−1^ as well as an excellent rate capability, delivering a capacity of 475 mAh g^−1^ even at 1000 mA g^−1^. This enhanced electrochemical performance of SiO_2_@C/MWNT ternary composite anode was associated with its unique core-shell and networking structure and a strong mutual synergistic effect among the individual components.

## Background

Due to its low lithium intercalation potential as well as excellent cycling performance, graphite has been widely adopted as a commercial anode for lithium-ion batteries (LIBs) [1]. Nevertheless, the theoretical capacity of graphite is only 372 mAh g^−1^, which cannot fulfill the ever-growing demands for high-performance batteries. Therefore, the development of next-generation anode materials with a larger specific capacity is necessary [2, 3].

Due to a large theoretical capacity of 1965 mAh g^−1^ and a low electrochemical potential, SiO_2_ is considered as a potential alternative to traditional carbonaceous anode materials. Furthermore, environmental friendliness, low cost, and natural abundance make SiO_2_ a commercial viable electrode material for LIBs. However, its practical application in LIB is commonly hampered by its poor electronic conductivity as well as a drastic volume variation upon charge-discharge process, resulting in particle pulverization and electrode deterioration with cycling [4–6].

One of the effective approaches to overcome these issues is to design SiO_2_-based composites by confining SiO_2_ particles inside conductive and flexible matrixes [7, 8]. In our previous study, Cu/carbon was introduced into the SiO_2_ composite as a dispersive matrix due to its good conductivity and effective buffering of the volume change of SiO_2_ [9]. It was shown by Yu et al. [10] that coating the SiO_2_ surface with carbon could be an efficient method to enhance its electrochemical performance, because such coating not only improves conductivity of the system but also accommodates the volume changes of the active material upon cycling.

Considering that the contact between SiO_2_@C particles is not good enough and the SiO_2_@C particles tend to agglomerate during charge/discharge [11] in this work, we report an effective and easy method to synthesize a core-shell SiO_2_@C anchored on MWNT via a sol-gel and pyrolysis route. In this composite, a carbon layer is homogeneously coated on the SiO_2_ particles, significantly improving the electronic conductivity of the system. Furthermore, formation of the 3D electron transportation pathways by a uniform dispersion of MWNT in the composite leads to outstanding electrochemical performance of the composite as an anode material for LIBs.

## Methods

Nine cubic centimeter of tetraethyl orthosilicate (TEOS) ((C_2_H_5_O)_4_Si ≥ 99.5%) and 9 cm^3^ HCl (0.1 mol dm^−3^) were dispersed in ethanol (16 cm^3^) and stirred for 30 min. Meanwhile, 4 g citric acid (C_6_H_8_O_7_ · H_2_O ≥ 99.5%) and 2.2 cm^3^ ethylene glycol (C_2_H_6_O_2_ ≥ 99%) were dissolved in deionized water (10 cm^3^), and then 1.9 g MWNT dispersion (9 wt%, MWNT aqueous dispersion, Timesnano, Chengdu) (mass ratio of Si and MWNT = 6.6:1) was added into this solution with gentle stirring for 30 min. The two resulting solutions were thoroughly mixed and transferred into an evaporating dish and dried at 55 °C for 10 h. The resulting product was heated under Ar atmosphere for 1 h at 1100 °C to obtain SiO_2_@C/MWNT composite. A reference SiO_2_@C composite without MWNT was obtained following the same preparation route.

The crystal structure of the samples was characterized by X-ray diffraction (XRD D8 Discover, Bruker) employing Cu Kα radiation. Raman spectra were conducted with Ar-ion laser of 532 nm using the Via Reflex Raman imaging microscope system. The structure and morphology of the SiO_2_@C/MWNT composites were studied using scanning electron microscopy (SEM, Hitachi S-4800) and transmission electron microscopy (TEM, JEOL 2100), respectively. Surface elemental analysis was conducted by an energy-dispersive X-ray spectroscopy (EDX) attached to the TEM apparatus. The content of amorphous SiO_2_ in SiO_2_@C/MWNT composite was estimated by using a thermogravimetric analyzer (STD Q-600) under N_2_ flow (30 ml min^−1^).

The working electrodes were prepared by coating a homogeneous slurry containing 80 wt% active material, 10 wt% acetylene black (MTI, 99.5%), and 10 wt% polyvinylidene fluoride (PVDF) (Kynar, HSV900) binder dissolved in 1-methyl-2-pyrrolidinone (NMP, Sigma-Aldrich, 99.5%) onto a copper current collector by a doctor blade, and further drying at 65 °C for 12 h in a vacuum oven. The resulting SiO_2_@C/MWNT and SiO_2_@C composite electrode was punched into circular disks with a diameter of 10 mm and a mass loading of ~4 mg cm^−2^. The coin-type cells with high-purity lithium metal as the counter electrode were assembled in a glove box (MBraun) filled with argon (99.9995%). Galvanostatic charge and discharge tests were conducted on a multichannel battery tester (Neware, BTS-5 V5 mA) with the potential range of 0.01–2.5 V vs. Li/Li^+^ at various cycling rates. The Versa STAT electrochemical workstation was used to conduct cyclic voltammetry (CV) tests between 0.01 and 3 V vs. Li/Li^+^ at a scanning rate of 0.1 mV s^−1^ and electrochemical impedance spectroscopy (EIS) measurements in a frequency range from 100 kHz to 1 mHz.

## Results and Discussion

**Fig. 1:**
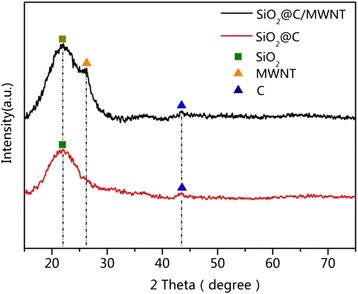
XRD patterns of SiO_2_@C and SiO_2_@C/MWNT composites

The phase purity of the SiO_2_@C/MWNT ternary composites was confirmed by XRD. It can be seen from Fig. 1 that in contrast with SiO_2_@C, the SiO_2_@C/MWNT composite shows a typical peak of graphitic carbon at 26.1°, indicating the presence of MWNT with the structure planes (200) [12]. A weak peak around 43° corresponds to a diffusion scattering of amorphous carbon coating, while a broad diffraction peak around 21° is associated with amorphous SiO_2_ [13, 14]. All the above results demonstrate that as designed, SiO_2_@C/MWNT ternary composite was successfully obtained.

**Fig. 2:**
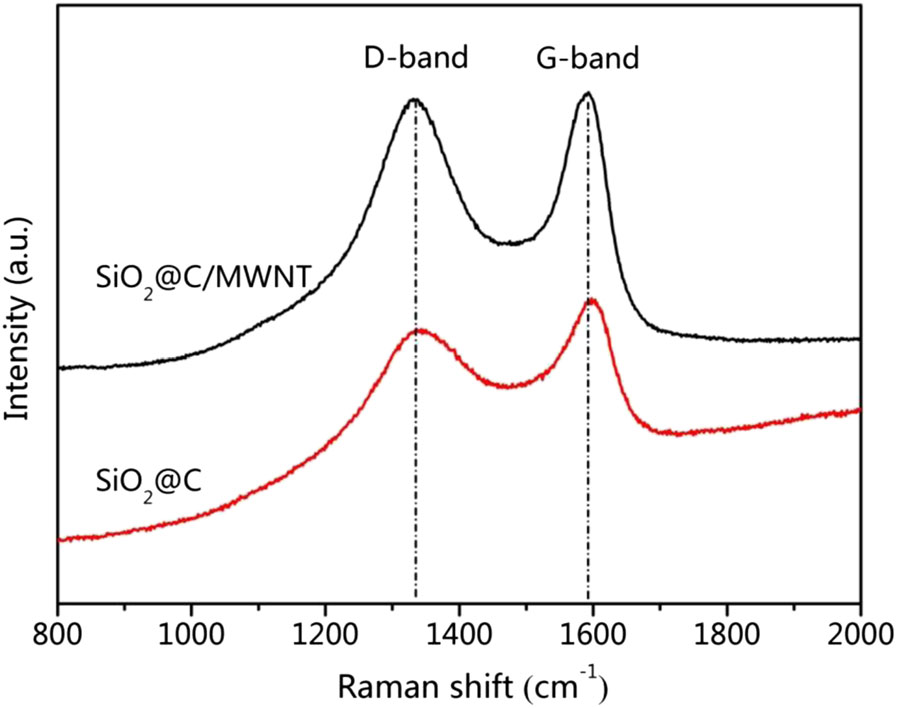
Raman spectra of SiO_2_@C and SiO_2_@C/MWNT composites

Raman spectroscopy was further performed to investigate the phase compositions in the SiO_2_@C/MWNT composite and the SiO_2_@C counterpart as shown in Fig. 2. Both samples possess double distinct peaks at 1340 and 1595 cm^−1^, related to the D and G bands of carbon, respectively [15]. These two vibration peaks demonstrate the low crystallinity of carbon [16]. The D band describes the defect-mediated zone-edge phonons and indicates the disordered carbon, edges, and defects, whereas the G band is a characteristic of the graphitic sheets, which according with the scattering of the E_2g_ mode apperceived for sp^2^ domains [17–19]. It is worth to note that the I_D_/I_G_ ratio for SiO_2_@C and SiO_2_@C/MWNT composites are 0.94 and 0.99, respectively. The I_D_/I_G_ of SiO_2_@C/MWNT composites increased compared with that of SiO_2_@C as a result of a strong binding interaction and the increased structural defects between Si and O [20, 21].

**Fig. 3:**
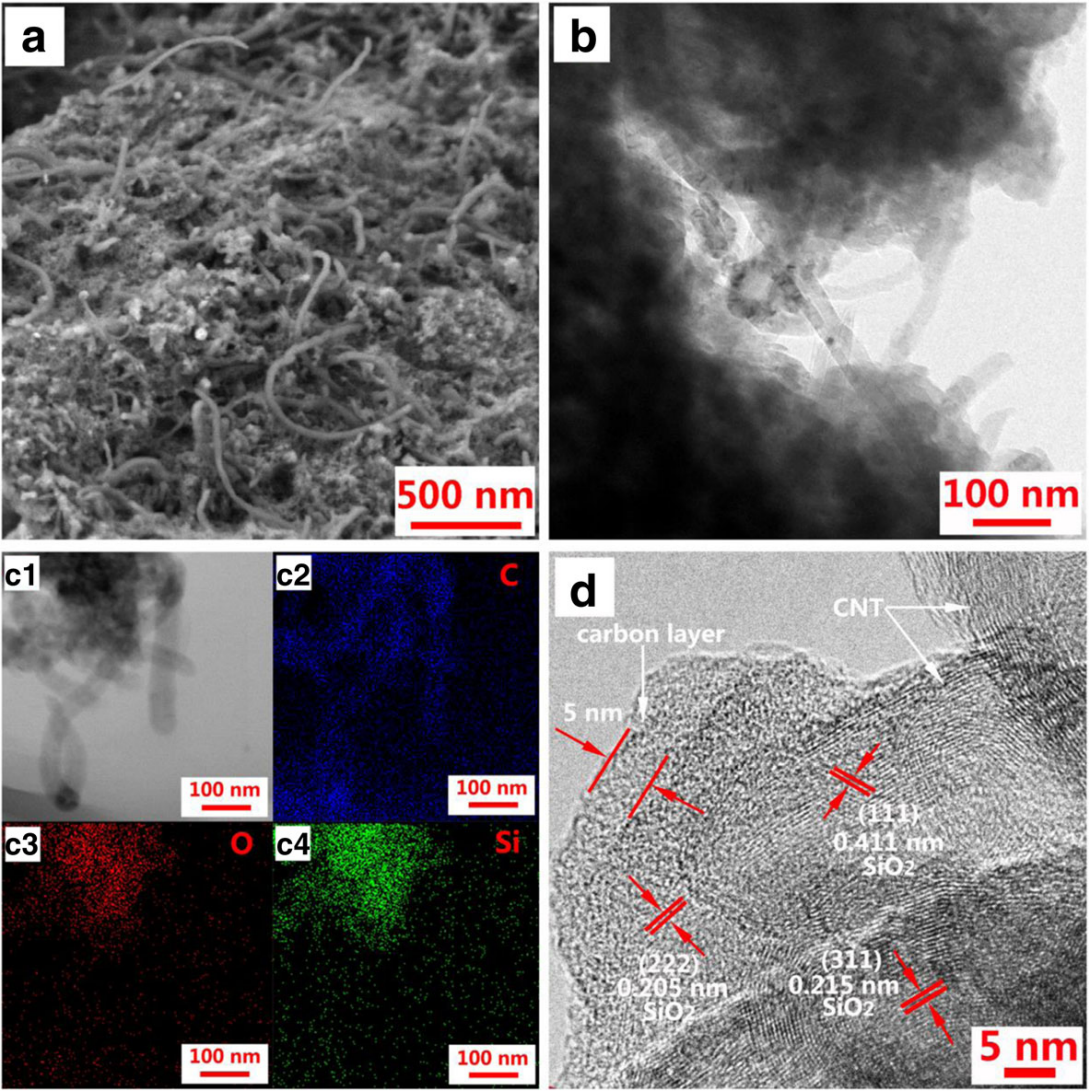
**a** SEM image and **b** TEM image of SiO_2_@C/MWNT composite. **c1** EDX mapping of C (**c2**), O (**c3**), and Si (**c4**) elements. **d** HRTEM image of SiO_2_@C/MWNT composite

As displayed in Fig. 3a, SEM confirms the micro/nano structure of SiO_2_@C/MWNT composite. The sample shows a disordered configuration with a wide size distribution. This could be considered as a verification of the amorphous structure of the material. From the TEM image (Fig. 3b), it can be seen that the MWNT-like bridges are directly connected to the SiO_2_@C particles, and this feature could support the structural integrity retention of the composite and favor the fast electron transfer. Meanwhile, MWNT of about 20–50 nm diameter intersperses among SiO_2_@C, which has an amorphous structure. The EDX element mapping (Fig. 3 (c1–c4)) indicates that the SiO_2_@C/MWNT composite contains homogeneously distributed O, Si, and C. One can see from Fig. 3d that an amorphous carbon layer with a thickness of about 2–7 nm is formed on the surface of SiO_2_. A turbostratic structure without crystalline lattice is discovered, indicating that the SiO_2_@C/MWNT composite has an amorphous structure. It is worth noting that MWNT is evenly distributed in the disordered matrix. A small amount of a microcrystalline structure domain could be observed in the composite, which lattice fringes with the spacing of about 0.205, 0.215, and 0.411 nm agree well with the spacing between (222), (311), and (111) of SiO_2_.

**Fig. 4:**
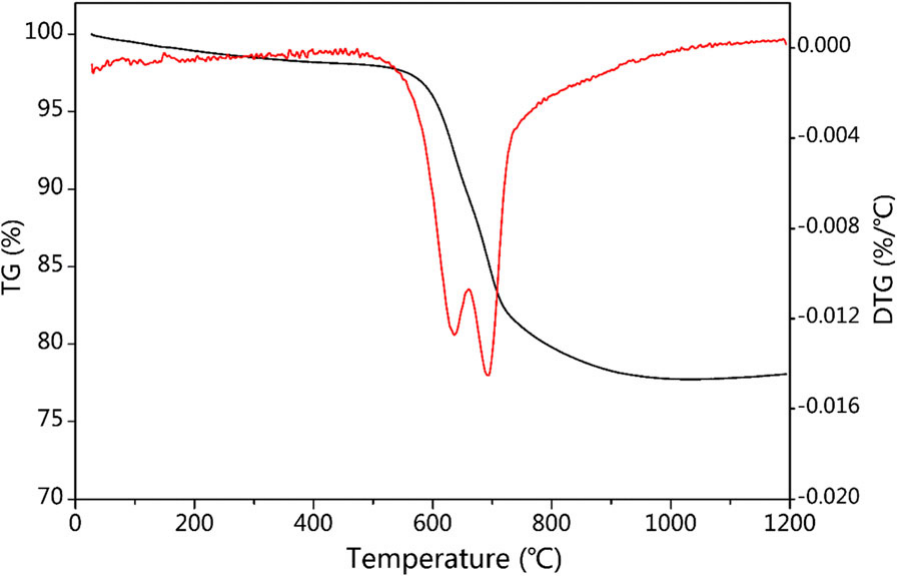
TG (*black*) and DTG data (*red*) of SiO_2_@C/MWNT composite

In order to verify the content of amorphous SiO_2_ in SiO_2_@C/MWNT composite, the TG and DTG data were collected and the results are shown in Fig. 4. The prominent weight loss between 550 and 730 °C, reflected in the TG curve, is related to oxidization of carbon and MWNT. Furthermore, the DTG curve shows two distinct peaks at 635 and 690 °C, which correspond to decomposition reaction of carbon layer and MWNT. Based on the positions of these two curves, the SiO_2_ content in the ternary composite can be estimated as ca. 77.5 wt%. Considering these data and the TG results, the mass composition of SiO_2_@C/MWNT could be estimated as SiO_2_:C :MWNT = 77.5:17: 5.5 wt%.

**Fig. 5:**
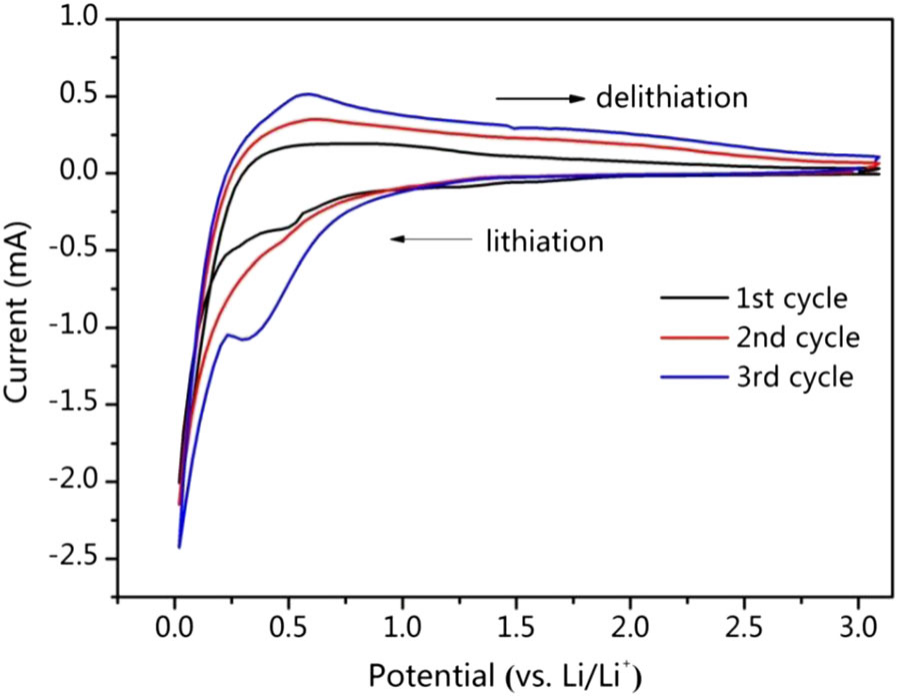
Cyclic voltammograms of SiO_2_@C/MWNT composite electrode at a scan rate of 0.1 mV s^−1^

The CR2025 coin cells were assembled to test the electrochemical performance of the SiO_2_@C/MWNT nanocomposite. Figure 5 shows the CV data of SiO_2_@C/MWNT. The CV curves present a reduction peak at approximately 0.57 V vs. Li/Li^+^ at the first cycle. It is related to the reduction reactions of lithium with SiO_2_ resulting in the side products of Li_4_SiO_4_, Li_2_Si_2_O_5_, and Li_2_O. Among these, Li_2_Si_2_O_5_, as reported, is active in the subsequent cycles, which enhances the electrochemical performance of the system [22], and Li_2_Si_2_O_5_ is reversible while the Li_2_O and Li_4_SiO_4_ phases are irreversible upon cycling. The increase of current in the CV curves could be related with this phenomenon. Along with this, this phenomenon could be considered as a part of electrochemical activation of the electrode upon its cycling, which is commonly observed for porous composite systems. A cathodic peak at 0–0.5 V can be observed in the initial cycle, corresponding to the alloying process of SiO_2_ [23]. On the other hand, the anodic peak at 0.24–0.9 V is extensive in the Li extraction part, matching well with the de-alloying process between amorphous Li-Si alloys and amorphous SiO_2_ [24, 25].

**Fig. 6:**
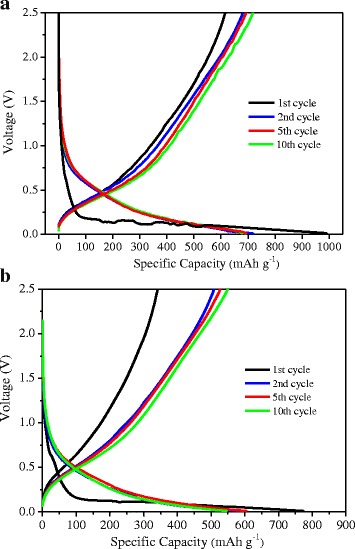
Charge/discharge profiles of **a** SiO_2_@C/MWNT and **b** SiO_2_@C composite electrode at a current density of 100 mA g^−1^

Figure 6a presents the charge/discharge curves of the SiO_2_@C/MWNT composite anode. The composite exhibits the initial discharge capacity of about 991 mAh g^−1^ while a corresponding charge capacity is about 615 mAh g^−1^, and this results in the initial coulombic efficiency of 62%. This relatively low coulombic efficiency could mainly be due to the formation of the solid electrolyte interface (SEI) on the electrode surface during the initial charge/discharge process. The discharge capacity becomes stable after 10 cycles, and the coulombic efficiency increases to ~100%. It is found that the charging potential profile is extraordinarily steep at potentials exceeding 1.4 V, which is due to a glassy state character of SiO_2_ with a strong polarization [26]. As shown in Fig. 6b, the potential profiles of the SiO_2_@C composite are similar to the profiles of the ternary composite but they exhibit lower capacities.

**Fig. 7:**
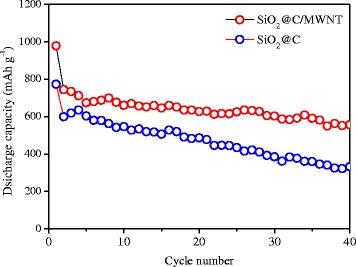
Cycle performance of SiO_2_@C and SiO_2_@C/MWNT composite electrodes at a current density of 100 mA g^−1^

**Fig. 8:**
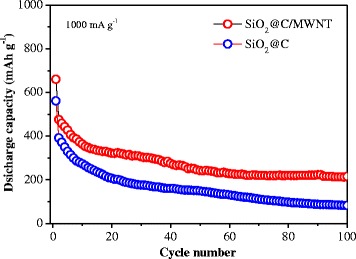
Rate performance of SiO_2_@C and SiO_2_@C/MWNT composite electrodes at a current density of 1000 mA g^−1^

The counterpart SiO_2_@C composite was tested in the same electrochemical environment. As shown in Fig. 7, the comparative cycling performance studies of the binary and ternary electrodes were evaluated at a current density of 100 mA g^−1^. It is obvious that the SiO_2_@C/MWNT sample shows remarkably enhanced cyclability than its SiO_2_@C counterpart. Specifically, the SiO_2_@C/MWNT exhibits a high specific capacity of 744 mAh g^−1^ at 100 mA g^−1^ in the second cycle and maintains a capacity of 557 mAh g^−1^ after 40 cycles. However, the corresponding capacity of SiO_2_@C retains only a capacity of about 333 mAh g^−1^ at the 40th cycle. The superior cycling stability of the SiO_2_@C/MWNT electrode could be attributed to the introduction of well-dispersed MWNT in the composite. Incorporation of MWNT with SiO_2_@C is designed to provide pathways for electrolyte/Li^+^ ingress and to accommodate the anode active mass volume expansion during cycling [27]. An outstanding rate capability of the SiO_2_@C/MWNT ternary electrode is illustrated in Fig. 8. One can see that after 100 cycles, the specific discharge capacity of the cell with the SiO_2_@C/MWNT composite cathode slightly decreases, and it exhibits a capacity of 215 mAh g^−1^ at a high-current density of 1000 mA g^−1^, presenting its enhanced electrochemical stability. In the same time, the SiO_2_@C composite retains a capacity of only around 95 mAh g^−1^ when cycled at the same current density.

**Fig. 9:**
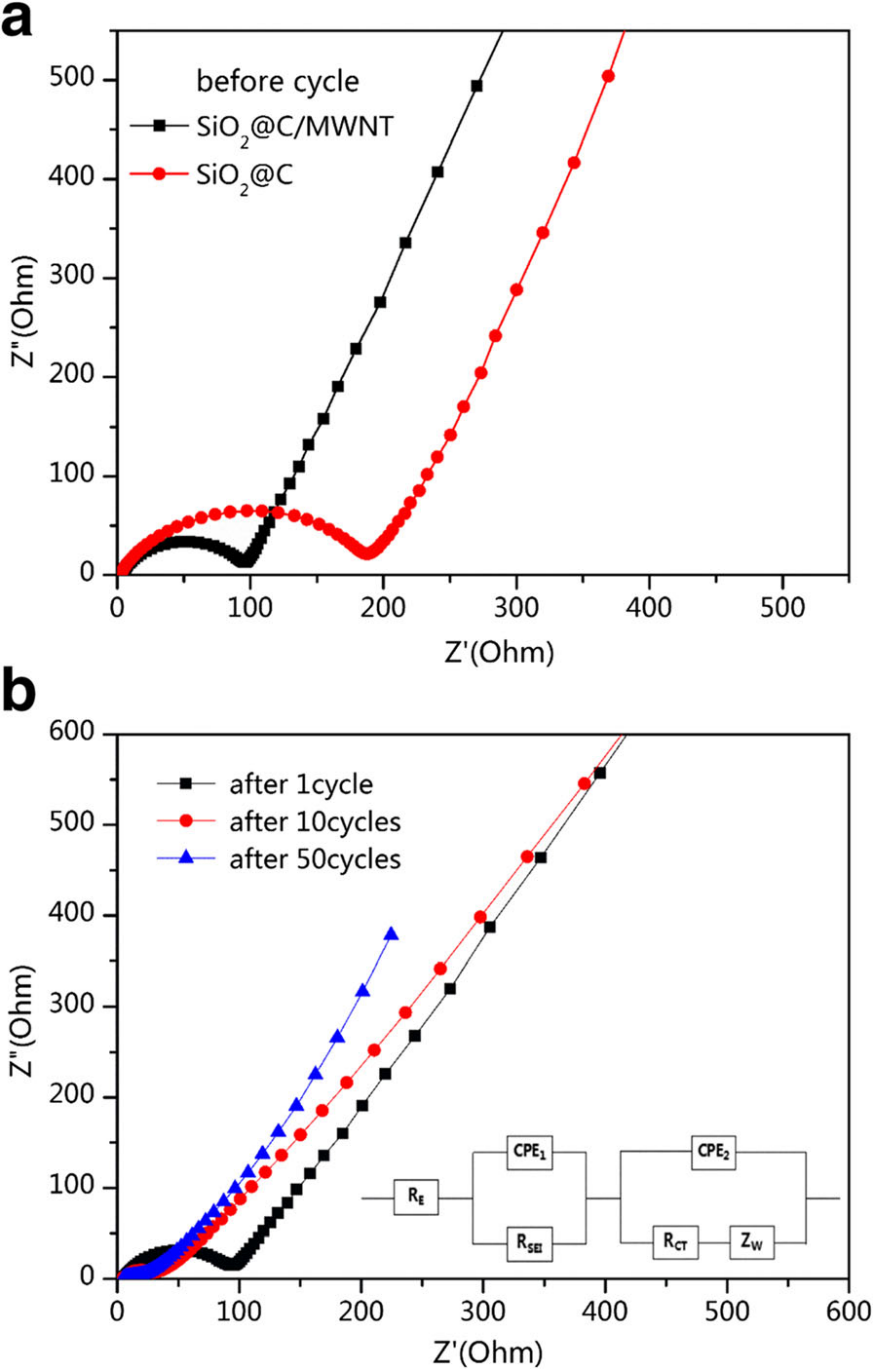
**a** EIS spectra of SiO_2_@C/MWNT and SiO_2_@C electrodes before cycling. **b** EIS spectra of SiO_2_@C/MWNT electrode upon cycling and an equivalent circuit obtained for this system

In order to further clarify the role of MWNT networks in the ternary composite, the EIS measurements were performed and the results are shown in Fig. 9. It can be seen that for the fresh cells, the diameter of the compressed semicircle in the high-to-medium frequency range for the SiO_2_@C/MWNT ternary electrode corresponds to 95 Ω, which is about half of that for SiO_2_@C, indicating that MWNT remarkably improves the conductivity and enhances the charge transfer properties of the ternary electrode. Figure 9b shows changes of EIS upon cycling and an equivalent circuit with a series of constant phase elements (CPE) and resistances obtained from the EIS data fitting. R_E_ reflect the bulk resistance of the electrolyte. The CPE_1_ and R_SEI_ are the charge capacitance and resistance of the solid electrolyte interphase (SEI) layer, respectively. The CPE_2_ and R_CT_ are related to charge-transfer, which mirrors the lithium ions intercalation into the electrode. The inclined line is generated by the Warburg impedance (Z_W_), which represents the lithium-diffusion process within SiO_2_@C/MWNT. After the initial cycle, the diameter of the semicircle remains the same at about 95 Ω but the slope of the Warburg component decreases compared with that of a fresh cell, reflecting the lithium-ion diffusion process within the electrode. Further, the resistance of the ternary electrode decreases to about 30 Ω due to the activation process. After 50 cycles, the diameter of the semicircle tends to stabilize, i.e., there is no remarkable impedance change, which evidences the stability of the ternary electrode upon cycling and its ability to be well adapted to the volume changes. These results confirm that MWNT can obviously improve the conductivity and enhance the structure stability of the SiO_2_@C/MWNT ternary electrode.

**Fig. 10:**
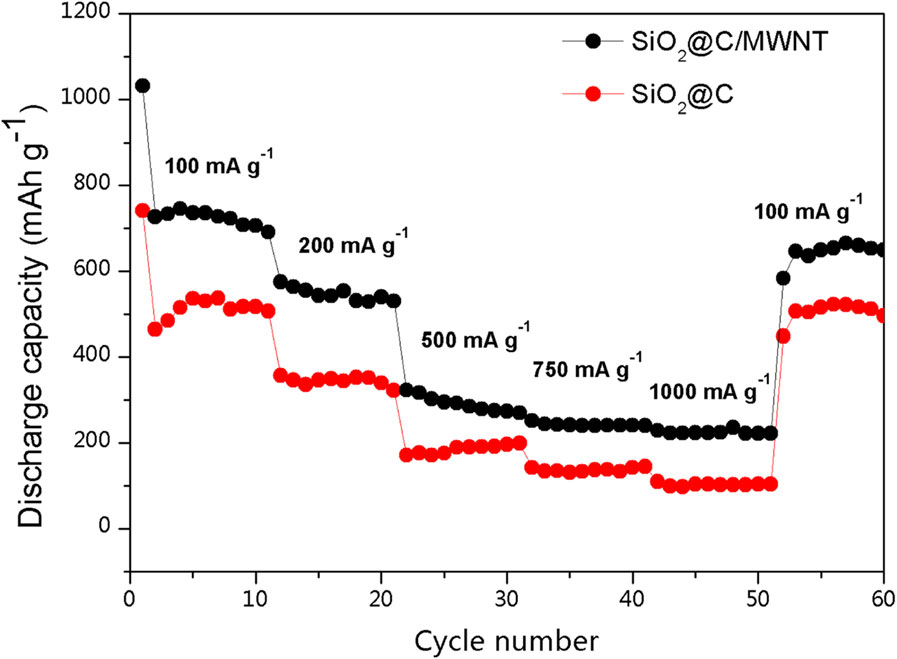
Rate performance of SiO_2_@C/MWNT and SiO_2_@C electrodes at various current densities

Furthermore the SiO_2_@C/MWNT nanocomposite electrode exhibits a good-rate capability as shown in Fig. 10. The SiO_2_@C/MWNT electrode delivers reversible capacities of ~710, 570, 300, 250, and 220 mAh g^−1^ at current densities of 100, 200, 500, 750 and 1000 mA g^−1^, respectively. When further, the current density was returned to 100 mA g^−1^, about 95% of the initial capacity could be recovered, indicating a good structural and electrochemical stability of the system. It can also be seen from Fig. 10 that the reversible capacities of SiO_2_@C are lower than that of SiO_2_@C/MWNT over a whole range of the current densities studied. It can be concluded that the MWNT component enhances the conditions for lithium-ion diffusion and the electric conductivity of the composite, favoring its rate capability.

Table 1 compares the performance data reported for the silicon anode for lithium-ion batteries with the results of this work. It can be seen that the SiO_2_@C/MWNT electrode prepared in this work exhibits an enhanced electrochemical performance compared with those reported previously. One can see that the reversible capacity and capacity retention of SiO_2_@C/MWNT at 40th cycles are higher than for most of other silicon electrodes reported in the literature. These results indicate that the SiO_2_@C/MWNT composite with a carbon containing layer structure and MWNT could be considered as a promising anode for high-performance Li-ion batteries.

**Table 1:** Performance comparison of SiO_2_ and SiO_2_@C electrodes for LIBs

Materials	Reversible capacity (mAh g^−1^)	Initial discharge/charge specific capacity (mAh g^−1^)	Current density	Cutoff potential range (*V*)	Ref.
Carbon-coated SiO_2_ nanoparticles	Above 500 (50th)	900/536	50 mA g^−1^	0.001–3	[10]
Silicon oxide-carbon	601 (100th)	1055/458	70 mA g^−1^	0.01–2	[28]
SiO_2_@GA composite	300 (110th)	1042.7/453.3	500 mA g^−1^	0.01–3	[29]
SiOx@C	630 (150th)	1160/820	50 mA g^−1^	0.01–3	[22]
Ag-deposited 3D porous Si	755 (50th)	1906/–	50 mA g^−1^	0.02–1.5	[30]
SiO_2_@C/MWNT	557 (40th)	991/615	100 mA g^−1^	0.01–2.5	This work

## Conclusions

The SiO_2_@C/MWNT ternary composite was successfully synthesized by a simple sol-gel method using low-cost citric acid and TEOS as starting materials, followed by heat treatment. Due to its unique core-shell and network structure and enhanced contact between its individual components, the resulting ternary composite cathode exhibited a remarkably enhanced electrochemical performance compared with the binary SiO_2_@C counterpart. Considering the simplicity and efficiency of the preparation process and outstanding electrochemical performance, the SiO_2_@C/MWNT composite can be considered as a promising anode material for the next generation lithium-ion batteries.

## Abbreviations

CV: Cyclic voltammetry
EDX: Energy-dispersive X-ray spectroscopy
LIBs: Lithium-ion batteries
MWNT: Multi-walled carbon nanotube
SEM: Scanning electron microscope
SiO_2_: Silica
SiO_2_@C: Carbon-coated silica composite
SiO_2_@C/MWNT: SiO_2_@C nanoparticles anchored on MWNT
TEM: Transmission electron microscope
TEOS: Tetraethyl orthosilicate
XRD: X-ray diffraction
